# Effects of Ultrafine Fly Ash against Sulphate Reaction in Concrete Structures

**DOI:** 10.3390/ma17061442

**Published:** 2024-03-21

**Authors:** Demet Demir Şahin, Hasan Eker

**Affiliations:** 1 Mining Technology Program, Department of Mining and Mineral Extraction, Gumushane University, 29000 Gumushane, Turkey; demetsahin@gumushane.edu.tr; 2 Property Protection and Safety Division, Occupational Health and Safety, Karabuk University, 78000 Karabuk, Turkey

**Keywords:** Blaine fineness, waste fly ash, cement, concrete, substitution rate, sulphate composition

## Abstract

In this study, Afşin Elbistan C-type fly ash (FA) was used, which protects against the sulphate reaction that damages concrete. The detrimental effects of post-reaction decrease with increasing fly ash fineness. The study used 10%, 30%, and 50% weight substitutes of cement. The fly ash was ground in a ball mill for 0, 10, 20, 30, 45, and 60 min, and Blaine fineness values of 1555, 1632, 2347, 2589, 2766, and 3433 cm^2^/g were obtained, respectively. The effect of the samples on the sulphate resistance was investigated by exposing the samples to 5% or 10% added sulphate solutions, and the compressive strength and ultrasonic pulse velocity of the concrete were tested. The compressive strength values obtained decreased with the increase in sulphate content, and the increase in the grinding time and the amount of substituted FA increased the compressive strength values. It was observed that weight loss increased with increasing sulphate content and decreased with the addition of FA with a high Blaine fineness. It was determined that as the Blaine fineness value increased, the sulphate content, FA substitution amount, and ultrasonic pulse speed decreased. This study was carried out to determine the effects of fly ash used at different fineness and replacement ratios on the performance and strength of concrete after exposure to external influences such as sulphate. The use of fly ash instead of cement will reduce the use of waste materials and natural resources and prevent environmental pollution. The cost of cement and concrete will be reduced.

## 1. Introduction

Concrete is a composite material formed by the combination of aggregate, cement, water, minerals and chemical additives. Concrete components can be produced anywhere, have a low energy consumption during manufacturing, and are the most frequently produced and consumed construction material due to their durability, economy, and ease of shape [1]. In particular, buildings such as schools, hospitals, roads, and bridges, which people use a lot, are expected to be designed to last a long time, be resistant to external effects, and outlast long-term damage. The destructive effects that impact these structures are based on their physical, chemical, and mechanical origins. Climate change contributes to a number of these issues, including solvents used to melt ice during the winter and physical deterioration brought on by high temperatures. Depending on the materials that make up the components of the concrete and the harmful substances that leak from the outside to the concrete, especially the alkali–silica reaction, sulphate effect, carbonisation, and corrosion, some acid and salt effects occur at the beginning of reactions that chemically damage the concrete. Finally, mechanical factors such as impact, abrasion, and erosion reduce the concrete’s strength over time [2,3,4,5,6].

Many parameters influence concrete strength and the damaging effects of sulphate. These parameters can be divided into internal and external factors. Internal factors, including concrete mix components and ratio, cement type, cement content, water/binding ratio, additives and materials used for cement, curing and handling, hardened concrete properties such as porosity, structure, permeability, distribution and mechanical properties, sulphate type and concentration, immersion type, and exposure temperature, are factors that determine the damaging intensity of sulphate in concrete [7,8,9,10]. Environmental conditions are the beginning of external factors that determine the magnitude of sulphate in concrete [10,11,12].

Concrete can be broken for various reasons, but irrigation due to sulphate is the main cause of premature concrete deterioration [1]. The degree of sulphate damage to concrete is caused by the soil used in concrete production, high clay soil, seawater, organic matter found in swamps, mines and sewage pipes, and rainfall [13,14,15]. The sulphate ions from these waters are accompanied by cations such as potassium, sodium, calcium, and magnesium [16,17]. The most damaging feature of these is magnesium sulphate, followed by sodium sulphate. These two sulphate contents are far more destructive than calcium, potassium, and ammonium sulphate [18]. Calcium sulphate from sulphate content has a shallow resolution, while sulphate damage is mainly caused by cement hydrates reacting with sodium sulphate to create magnesium sulphate [19,20,21,22,23].

Thanks to mineral additives, it is also possible to increase the strength of concrete against sulphate damage and to recycle the waste material released into nature [24,25]. Waste recycling and pollution in the environment, as well as waste destruction in the environment, are significantly reduced [26,27,28,29,30,31,32]. Samples prepared with fly ash used in concrete instead of cement are preferred due to the reduced compressive strength losses compared to standard concrete [33,34,35,36]. In addition, sulphates are prevented from reacting by connecting the Ca(OH)_2_ in the fly ash environment to make the concrete more durable against sulphates found in the environment. This reduces the binder’s Ca(OH)_2_ and C3A ratios [37,38,39]. These two harmful chemical reaction products are the result of sulphate ions combined with aluminium (C3A) and calcium (Ca(OH)_2_) components in hardened concrete [40,41,42,43]. The expansion of these components in concrete affects the formation of cracks, the increase in permeability, and the adherence of aggregate–cement dough, causing the concrete to ultimately deteriorate [44,45].

Sulphate ions in concrete cause chemical reactions and disturbances. The most effective method to reduce or avoid the harmful components of the chemical components of cement is to replace them with other materials [46,47,48]. Work is being undertaken to minimise or eliminate the damaging effects of sulphate [49]. In these studies, mineral additives such as fly ash, silica fume, and metakaolin were concentrated to try to increase cement’s sulphate resistance [50,51]. Because such waste materials improve the properties of the concrete’s porous structure, increase its resistance to chemical damage, neutralise the chemical solution, and accumulate products that react, the destructive structure becomes passive [52].

In this part, fly ash fineness is activated and contributes to the thin porosity of the concrete and greater strength after a chemical attack [53]. In Ouyang et al.’s [54] study, concrete produced with a fine fly ash contribution reduced the porous volume compared to concrete with thick fly ash additives. They have shown that fly ash can be shredded to have a high degree of fineness and that it can be used as a concrete material [25,44,45,46,49,50,51,52,55,56,57,58]. In another study, the fly ash used as a thin material in concrete increased the durability of the concrete, reduced the number of pores, and reduced the chloride penetration resistance and sulphate damage due to an increase in strength [59]. In the study conducted by Chindaprasirt et al. [13], they said that using F-type fly ash reduces expansion, primarily by increasing the fineness of the fly ash to increase the resistance against sulphate. In addition to the importance of the environmental and economic impact of fly ash, its use as a cement replacement contributes greatly to saving resources and improving concrete performance. In addition, it prevents sulphate damage and microstructural deterioration of the concrete produced [39,60].

In this study, six different fineness values of fly ash and three different substitution ratios were used in concrete composition instead of cement on a weight basis. The effect on the performance of fly ash-admixed concrete was investigated by exposing the concrete samples to different sulphate solutions.

### Importance and Purpose of the Research

Many factors contribute to the damaging effect of sulphate in concrete. For example, concrete exposed to sulphate causes premature deterioration. The widespread use of fly ash to increase concrete’s resistance to wear and tear, external effects, and damaging elements has been explored and discussed in many studies. The unique aspect of this study compared to other studies is that when concrete is created with differing amounts (10%, 30%, and 50%) of cement displacement by fly ash ground to different finenesses for 0, 10, 20, 30, 45, and 60 min in a ball mill and exposed to 5% or 10% sulphated water, it has been found to be resistant to sulphate. It was understood that the fineness of sulphate versus fly ash has a filler effect, preventing harmful sulphate compounds from passing through the gap solution. The slimness increase and pozzolanic activity are connected, and harmful sulphate compounds are removed from the environment. Strength values were determined by compressive strength testing of the fly ash fineness and the substitution ratio against sulphate in concrete. The test revealed that fine and substituted concrete samples showed better resistance to sulphate. In addition, weight loss and ultrasonic pulse velocity experiments were applied to the samples, and the values obtained were compared with the compressive strength values. 

## 2. Materials and Methods

The materials used in the concrete samples produced in the study are limestone aggregate, cement, fly ash, and tap water.

### 2.1. Cement

The study used CEM I 42.5 cement from the cement factory (Gümüşhane-Aşkale Cement Plant, Gümüşhane, Turkey). Table 1 lists the cement’s chemical properties.

### 2.2. Fly Ash

The study employed fly ash from the Kahramanmaraş Afşin Elbistan thermal power plant (Afşin Elbistan thermal power plant, Kahramanmaraş, Turkey). Fly ash from Afşin Elbistan was classified using the ASTM C 618 [61] standard. It was categorised as a fly ash class C object. In order to ascertain the physical characteristics of Afşin Elbistan fly ash, the laboratory of the Gümüşhane Aşkale Cement Factory, Turkey carried out the necessary investigations (Table 2).

### 2.3. Aggregate

**Figure 1:**
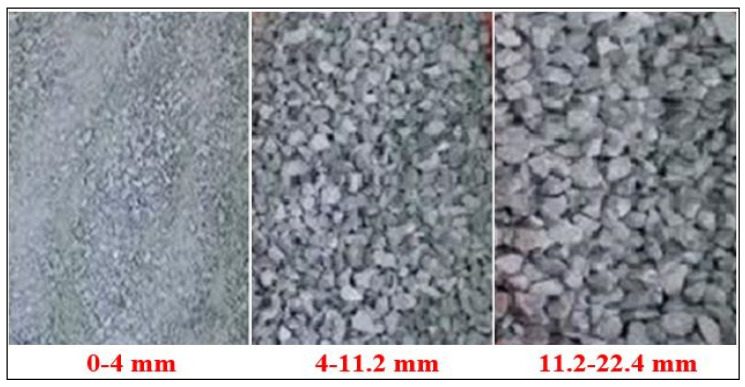
Limestone aggregate from the Gümüşhane region with different grain sizes in fly ash-reinforced concrete production.

**Figure 2:**
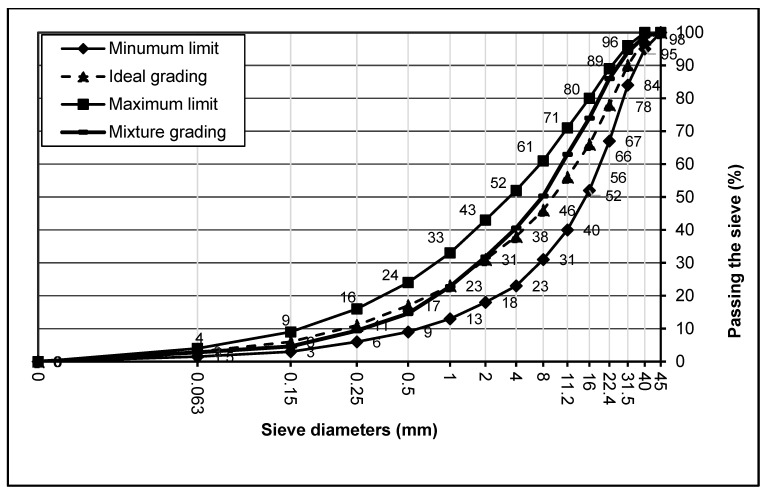
Gradation curve of combined aggregate used in the concrete mixture by TS802.

The aggregate used in the study was limestone from the quarry used by Gumushane concrete plants. Different grain sizes of this aggregate were used in the concrete blends created within the scope of operation. The different dimensions used in these concrete mixtures were 0–4, 4–11.2, and 11.2–22.4 mm, as shown in Figure 1. In addition, Figure 2 shows the grain size curve expressed in the standard TS 802 [62].

### 2.4. Mixing Water

The mixing water used to prepare the concrete samples was tap water from the Gümüşhane area.

### 2.5. Method

The concrete produced in the study has a C25 strength class. The concrete produced according to this strength class was kept in water-filled curing pools for 28 days, and the final strength of the concrete was achieved. Concrete samples were then exposed to water with 5% or 10% sulphate content, and weight loss, ultrasonic pulse velocity, and compressive strength values were determined. Different samples were used for 5% and 10% Na_2_SO_4_ solutions. For the fly ash fineness used in each solution, 36 specimens and 3 reference concretes, for a total of 39 concrete samples, were produced and subjected to experiments.

### 2.6. Specific Surface Area

The study’s values for specific surface areas are based on the TS EN 196-6 [63] standard. The obtained Blaine values were made using an automated Blaine device at the Gumushane Askale Cement Factory Laboratory (Table 3).

### 2.7. Preparation of Concrete Mixtures

Prepared concrete samples were produced at the Gümüşhane University Construction Engineering Building and Materials Laboratory (Table 4). The manufactured concrete is designed according to the C25 strength classes in the standard TS 802 [62]. The air content of the prepared concrete mixes was set at 20% and the density at 2690 kg/m^3^. In addition, the capacity of the mixer used was 100 L (the used volume was 56 L). 

**Figure 3:**
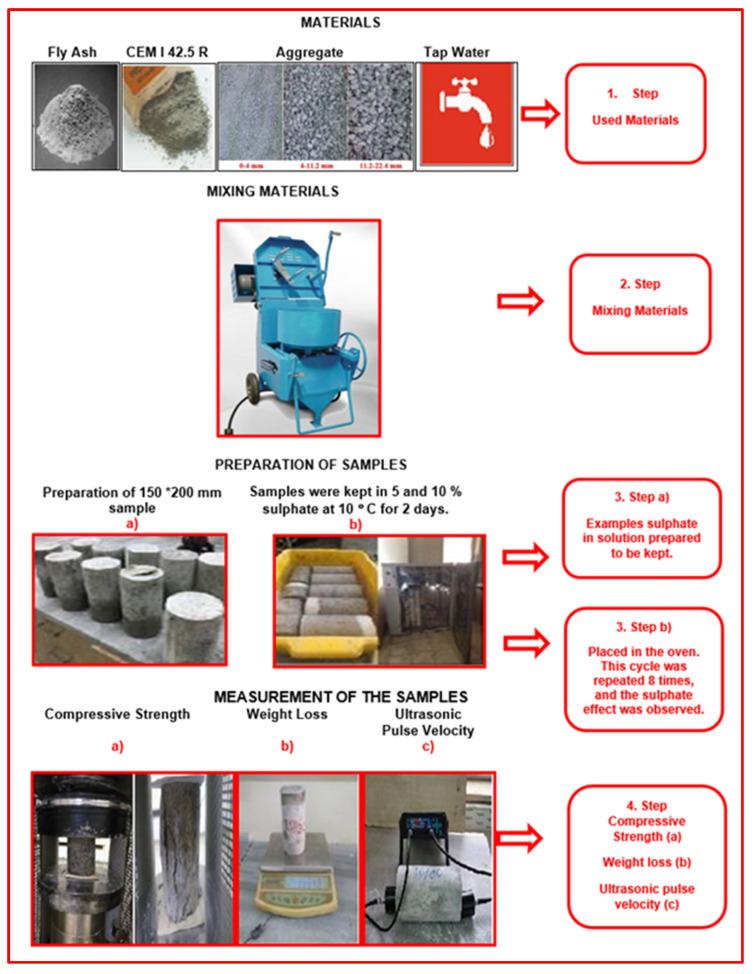
Flow chart of the sulphate resistance test.

The sulphate resistance wet–dry cycle according to ASTM C 1012 [7] standard was tested to determine the sulphate resistance of the manufactured concrete samples. The solution of sodium sulphate (Na_2_SO_4_) used in experiments was a 10% solution with 90 L of water for 100 L solution and a weight of 10 kg Na_2_SO_4_, and a 5% solution with 95 L of water for 100 L solution and a weight of 5 kg Na_2_SO_4_. At 105 °C, sodium sulphate (Na_2_SO_4_) was held for two days in a solution with 5% and 10% content after two days of standby. This way, a wet–dry cycle was performed eight times. After each cycle, the ultrasonic pulse velocity and weight losses of the concrete samples were determined. The current diagram for determining sulphate resistance is given in Figure 3.

### 2.8. Test of Ultrasonic Pulse Velocity

The prepared concrete samples were determined according to the ultrasonic pulse velocity measurement ASTM C597 [64]. It is derived from measuring the speed of the vibrational energy sent over the sample. After the horizontal hold of the concrete sample, the value of supersonic waves sent from one surface to another is measured and determined using Equation (1), which passes the sample to another surface.

V = (S/t)(1)

where V = P pulse velocity (meters per second), S = distance between the surface of the sample being sent over the sound wave and the surface of the wave being picked up (meters), and t = time from the concrete surface P to the surface where the wave was sent and received (microseconds).

### 2.9. Test of Weight Loss

The prepared concrete samples were extracted after being held in the curing pool for 28 days, and the samples were kept dry for one day in the laboratory environment. Dry samples are first weighed, and the initial weight value is noted. The samples were then held in the Na_2_SO_4_ solution with 5% and 10% content, and after eight cycles, the cycle was stopped. The concrete specimens were utterly deformed. The weight values of each post-cycle sample were measured, and the amount of change was expressed in % in Equation (2) by removing it from the initial weight value.

% Weight loss = [(Last Weight − First Weight)/First Weight] × 100(2)

### 2.10. Test of Compressive Strength

After the wet–dry cycle was completed, samples were subjected to compressive strength according to TS EN 12390-3 [65]. Three samples were broken at each grinding time when compressive strength values were determined, and the tip was averaged as a value.

## 3. Results

**Figure 4:**
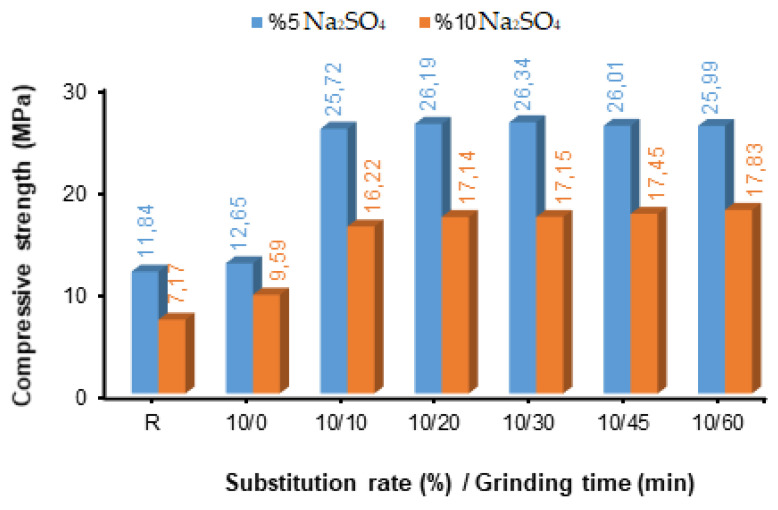
Compressive strength values of concretes with different fineness values and 10% fly ash replacement.

**Figure 5:**
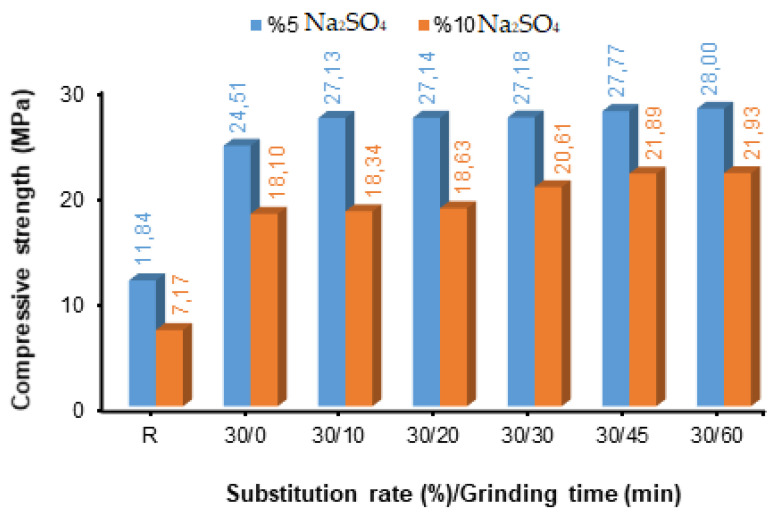
Compressive strength values of concretes with different fineness values and 30% fly ash substituted.

**Figure 6:**
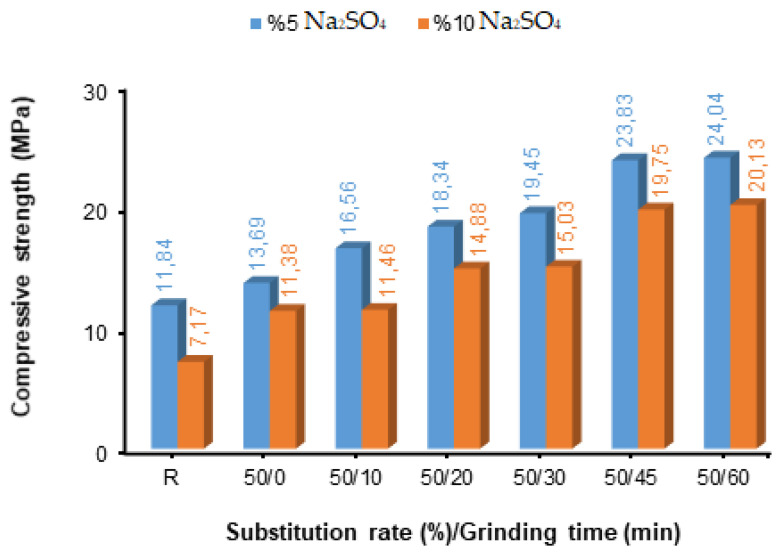
Compressive strength values of concretes with different fineness values and 50% fly ash replacement.

After grinding times of 0, 10, 20, 30, 45, and 60 min of fly ash (FA), blended concretes were produced by substituting fly ash of different fineness values in cement by 10%, 30%, and 50%. The compressive strength values determined after the concretes were produced were kept at 5% and 10% Na_2_SO_4_ content after 28 days of curing, as shown in Figure 4, Figure 5 and Figure 6.

Figure 4 shows that the compressive strength values of 10% fly ash-added concrete samples in a 5% Na_2_SO_4_ solution are higher than those of the samples kept in a 10% Na_2_SO_4_ solution. As the fly ash fineness of the samples in both solutions increased, the compressive strength values also increased. In Figure 4, graphs showing the changes in grain size of fly ash with grinding and the relationship between grinding times and compressive strength were created instead of Blaine values representing them in quantity. Since the obtained compressive strength values will create the same graphical display by writing the Blaine fineness values instead of the grinding times, no graphs are shown with the Blaine values. This situation is possible to see in Figure 5 and Figure 6. In addition, the compressive strength values of the fly ash-added concrete samples were higher than those of the reference concrete samples. Among the samples exposed to 5% and 10% sulphate solutions, the best strengths were the FA-substituted concrete sample with a fineness of 30 min grinding (26.34 MPa) and the FA-substituted concrete sample with a fineness of 60 min grinding (17.83 MPa), respectively (Figure 4). Along with 20 min of grinding time, it was understood that the compressive strength values were close in both solution ratios and that there was not much change in the compressive strength.

As seen in Figure 5, the compressive strength values increased when increasing the fly ash replacement ratio from 10% to 30%. Additionally, the compressive strength values of the fly ash-added concrete samples were higher than those of the reference concrete samples. Among the specimens exposed to 5% and 10% sulphate solutions, the best strengths were observed for the FA-substituted concrete specimen with a grinding fineness of 60 min (Figure 5). Along with 10 min of grinding time, it was understood that the compressive strength values increased slightly depending on the grinding time at the 5% solution ratio, while they increased greatly depending on the grinding time at the 10% solution ratio.

As a result of using a 50% fly ash replacement ratio instead of cement in the concrete composition, the compressive strength values decreased. Concrete samples with 50% FA replacement had lower compressive strength values than samples with 10% and 30% replacement ratios. However, despite the decrease in compressive strength, higher compressive strength values were obtained than in the reference sample. The best strengths were obtained from the samples exposed to 5% and 10% sulphate solutions: the 60 min grinding fineness in the FA-substituted concrete sample (24.04 MPa) and the 60 min grinding fineness in the FA-substituted concrete sample (20.13 MPa), respectively (Figure 6). Between 10 and 45 min of grinding time, the compressive strength values at 5% and 10% solution ratios continuously increased depending on the grinding time. It was observed that a 50% substitution of FA in concrete reduced the compressive strength values. However, an increase occurred between the fineness increase and the compressive strength values (Figure 6).

In this study, among the fly ash-added concrete exposed to 5% and 10% Na_2_SO_4_ solutions, the highest compressive strength values were found in the concrete samples with 30% FA replacement. However, fly ash fineness had a positive effect on all substitution rates and increased the strength. As a result, it has been understood that the FA replacement rate that can be used in the concrete composition is 30%. The compressive strength results obtained in the study are similar to those of other studies that support this situation. Fournier et al. [66] and ASTM C 618-22 [61] found that using a 30% fly ash substitute instead of cement in concrete blocks increases pozzolanic activity and supports it with the products it creates. The use of fly ash up to 20–30% in concrete, especially instead of cement, has been accepted as the optimum ratio in many countries. Many studies have demonstrated the effect of this ratio, as the widespread adoption improves the workability and long-term mechanical and durability properties of concrete due to the spherical shape and pozzolanic properties of fly ash [67,68,69,70]. Tkaczewska and Małolepszy [71] used fly ash samples with different fineness values to produce cement. Blaine fineness of cement was determined at 350 m^2^/kg, and Blaine fineness of fly ash was determined at 440 m^2^/kg, 320 m^2^/kg, 500 m^2^/kg, 350 m^2^/kg, 510 m^2^/kg, and 360 m^2^/kg, respectively. They determined the compressive strength values by substituting the fly ash with these fineness values in cement at a rate of 40% and keeping the mixtures they prepared in Na_2_SO_4_ solution for 90, 180, 365, and 730 days. The highest compressive strength values were obtained at 500 m^2^/kg Blaine fineness values, valid for all curing times. Sinsiri et al. [72] prepared mortar samples by using fly ash with three different Blaine fineness values (3215, 4440, and 5890 cm^2^/g), each fineness value being 0, 20, 30, and 40% instead of cement Type I and V. The prepared samples were subjected to a curing period of 1 to 540 days in a 5% magnesium sulphate solution. Using Portland cement type V with fly ash produced less expansion than that of Portland cement type I with the same level of fly ash fineness; mortar rods made with 40% fly ash substitution expanded less than 20% and 30% substitution. Mortar sticks with 20–25% fine fly ash replacement (Blaine 4440 or 5890 cm^2^/g) and mortar sticks with 40% original fly ash replacement (Blain fineness 3125 cm^2^/g) have the same expansion. The smaller particle size of fly ash produces not only fly ash mortar sticks but also a higher strength activity index than larger particle sizes, thus demonstrating the effective usability of very fine fly ash in reducing and improving expansion. Arel and Shaikh [73] used nano-silica (NS) and fly ash with three different fineness values (original, 2320 cm^2^/g, and 5980 cm^2^/g Blaine fineness values) in their study. In the study, mortar samples were prepared using 3600 cm^2^/g Blaine fine cement and 5%, 15%, and 60% fly ash instead of cement. They evaluated the effects on the mechanical and durability properties of the two types of fly ash mortars by exposing these samples to two types of curing and sulphate solutions. They determined that wet-cured mortars containing finer fly ash exhibited higher compressive strength at all ages than mortars containing thicker fly ash. Chindaprasirta et al. [13] investigated the effect of fly ash fineness on water requirements and some properties of hardened mortar. In addition to the original fly ash, five different Blaine fineness values of fly ash were obtained using a sieving and air separator: 3000 cm^2^/g for the original fly ash, 3900 cm^2^/g for the fly ash passing through the no. 200 sieve, 4800 cm^2^/g for the no. 325 sieve, 9300 cm^2^/g for the fine fly ash, 4900 cm^2^/g for the medium fly ash, and 1800 cm^2^/g for the coarse fly ash. These fly ashes were used at a rate of 40% instead of cement, and the compressive strength values were determined after 3, 7, 28, and 90 days of curing. The best compressive strength values were observed at all curing times in fly ash-added mortars and fly ash additives, which had the highest fineness value among these mortars. A significant improvement in resistance to sulphate expansion was obtained for all fineness values except coarse fly ash, where greater expansion was observed. Along with the fly ash’s fineness values, resistance to sulfuric acid damage also improved. The fact that the fly ash used was in coarse- and fine-grained structures contributed to the increase in the performance of the mortar with different properties. The coarse-grained fly ash additive binds the cement matrix better, reducing the losses in the products formed due to hydration. Due to the increase in the fineness value of fly ash with a fine-grained structure, the pozzolanic reactivity is higher, which makes the cement matrix more dense, resulting in better mechanical properties.

### 3.1. The Values of Weight Loss of Sulphated Concrete

**Figure 7:**
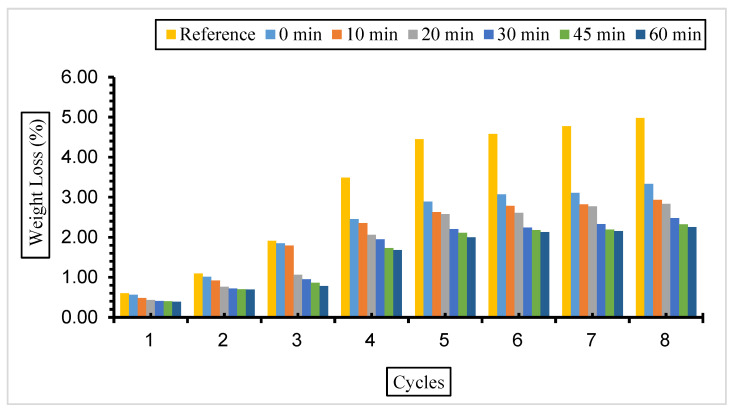
Weight loss values of concrete with different fineness values and 10% fly ash substitution in a 5% Na_2_SO_4_ solution.

**Figure 8:**
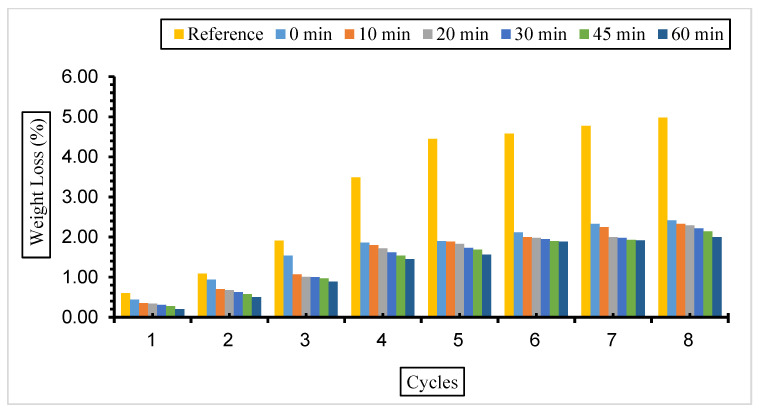
Weight loss values of concrete with different fineness values and 30% fly ash substitution in a 5% Na_2_SO_4_ solution.

**Figure 9:**
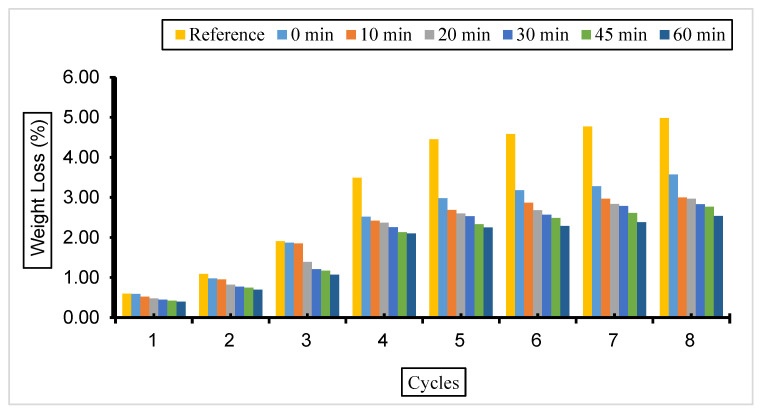
Weight loss values of concrete with different fineness values and 50% fly ash substitution in a 5% Na_2_SO_4_ solution.

Figure 7, Figure 8 and Figure 9 show the weight loss values of concrete with fly ash samples in a 5% Na_2_SO_4_ solution over eight cycles with a grinding time of 0, 10, 20, 30, 45, and 60 min across the cement and a 10%, 30%, or 50% substitution ratio.

The weight loss was most observed in the reference concrete without fly ash substitution compared to the concretes with different substitutions and grinding periods exposed to a 5% Na_2_SO_4_ solution. The weight loss decreased with the increase in fly ash fineness in the concrete samples prepared at all substitution rates. The highest weight loss was observed in concrete with 50% fly ash substitution, and the lowest weight loss value was observed in concrete with 30% fly ash admixture.

The weight loss values of the concrete exposed to a 5% Na_2_SO_4_ solution are in parallel with the compressive strength values, and the lowest weight and highest compressive strength values were observed in concretes with a 30% fly ash admixture.

**Figure 10:**
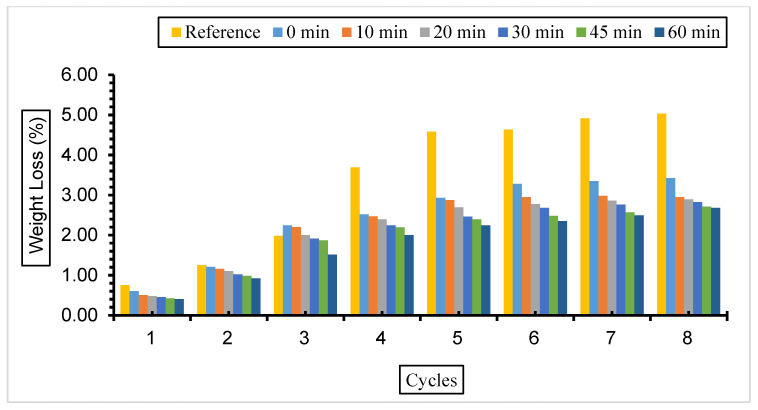
Weight loss values of concretes of different fineness values and 10% fly ash substituted in a 10% Na_2_SO_4_ solution.

**Figure 11:**
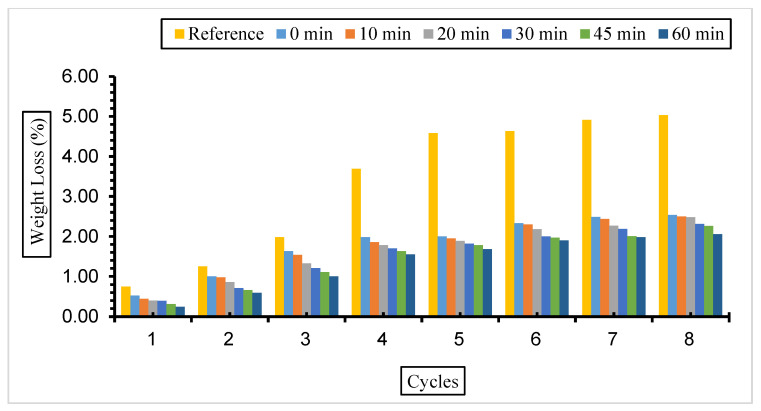
Weight loss values of concretes of different fineness values and 30% fly ash substituted in a 10% Na_2_SO_4_ solution.

**Figure 12:**
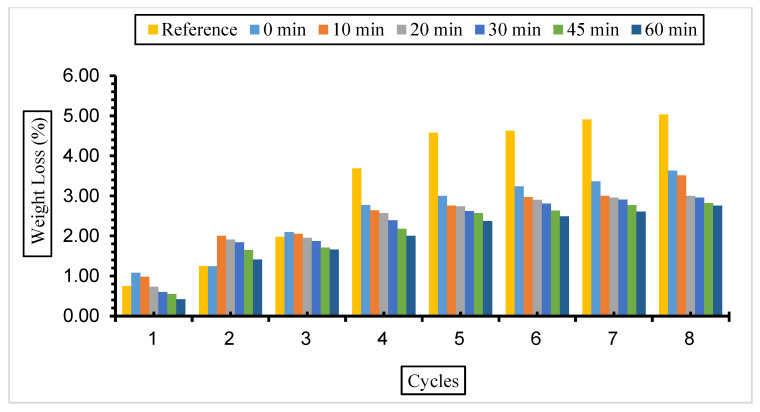
Weight loss values of concretes of different fineness values and 50% fly ash substituted in a 10% Na_2_SO_4_ solution.

Weight loss values for concretes with different replacement ratios and grinding times exposed to a 10% Na_2_SO_4_ solution are given in Figure 10, Figure 11 and Figure 12.

The weight loss values of the samples in this solution were similar to those in a 5% Na_2_SO_4_ solution. It was determined that all samples of fly ash-added concrete had less weight loss than the reference concrete. However, the minimum decrease in the fly ash substitution rate in concrete was 30%, and the highest weight loss was found in concrete with a 50% replacement rate. It was also determined that the amount of weight loss increased as the number of cycles increased but decreased with the increase in fly ash fineness.

According to the results obtained, it was determined that the weight loss value in concretes with different fineness values and substitution ratios kept in 5% and 10% Na_2_SO_4_ solutions decreased with fly ash fineness, and the minimum weight loss was from 30% substituted concretes. Binici et al. [74] stated in their study that the mass loss is less in samples with fly ash replacement than in reference concrete. Balakrishnan and Awal [75] investigated the durability of concrete using high volumes of fly ash in concrete. It was determined that the weight loss of concrete exposed to sulphate increased over time, but the weight loss decreased with the fly ash replacement ratio increase. The lowest weight loss was observed in the concrete with 60% fly ash. Alnkk [76] obtained the lowest weight loss in 0%, 5%, 10%, and 15% fly ash substitutions in concrete with a 15% admixture.

The reasons for the reduction in weight loss when exposed to sulphate with the use of fly ash in concrete will reveal more beneficial hydration products as the fly ash exhibits pozzolanic properties, and this property increases with fineness. Along with the increase in fly ash fineness, fine-grained fly ash fills the voids in the concrete better, forming compact concrete. Additionally, fly ash reacts with harmful products formed from hydration, eliminating these products and creating a concrete structure that is more resistant to sulphate damage. In addition, by producing more durable concrete with fly ash additives, mass losses that may occur in the structure of concrete are minimized [77]. Rozario et al. [78] prepared concrete samples using fly ash at 10%, 15%, 20%, and 25% in M20, M30, M40, and M50 coded concrete samples with different grain sizes. These specimens were exposed to a 5% sodium and magnesium solution for curing times of 28 and 56 days, respectively. Weight loss in concrete exposed to these solutions decreased depending on different concrete types, fly ash replacement rates, and increased curing time.

Along with the results obtained, the different types of concrete produced have reduced the harmful losses caused by fly ash. Especially in the concrete produced using aggregates of different sizes and the use of fly ash, the damaging factors due to expansion in concrete samples exposed to sulphate, the structure and number of cracks, as well as the loss of mass, were reduced, thus enabling the concrete to gain a more durable structure. In another study, concrete samples prepared with recycled aggregate, 10% silica fume, or a 30% fly ash additive were exposed to a 3.5% sodium sulphate solution. They determined that weight loss in fly ash-added concrete decreased eight times compared to standard concrete. Especially with the use of mineral additives (fly ash, silica fume, etc.) instead of cement in concrete, a tight pore structure is formed, and thus a decrease in the number of pores occurs. Whether exposed to sulphate originating from groundwater or water with a high sulphate content, the strength of the concrete makes it more resistant to these solutions. Concrete that provides high performance over a longer period of time contributes to the concrete industry’s sustainability [79].

### 3.2. The Values of Ultrasonic Pulse Velocity of Sulphated Concrete

**Figure 13:**
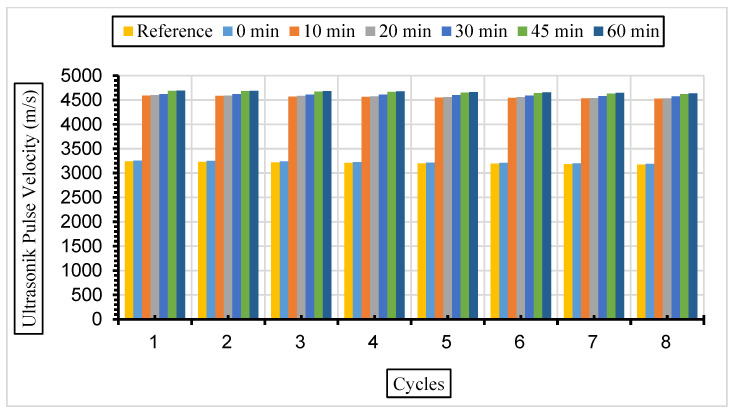
UPV values of concretes of different fineness values and 10% fly ash substituted in a 5% Na_2_SO_4_ solution.

**Figure 14:**
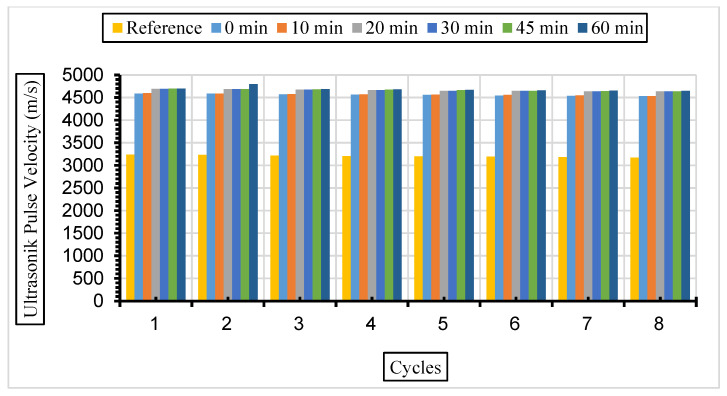
UPV values of concretes of different fineness values and 30% fly ash substituted in a 5% Na_2_SO_4_ solution.

**Figure 15:**
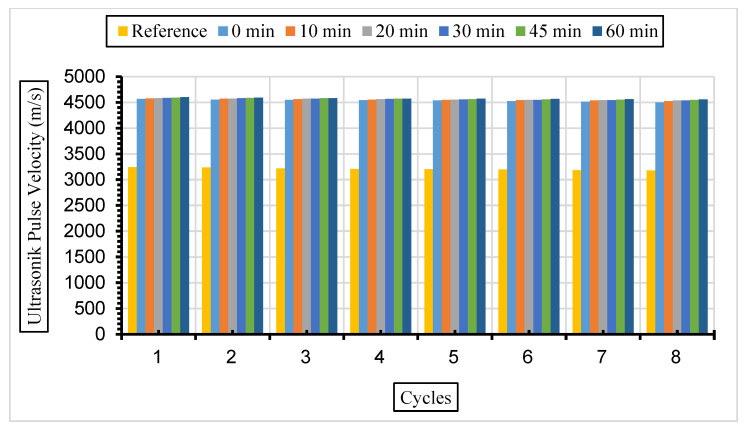
UPV values of concretes of different fineness values and 50% fly ash substituted in a 5% Na_2_SO_4_ solution.

Figure 13, Figure 14 and Figure 15 show the ultrasonic pulse velocity (UPV) values of the concrete samples exposed to a 5% Na_2_SO_4_ solution during eight cycles with fly ash with a grinding time of 0, 10, 20, 30, 45, and 60 min and a 10%, 30%, or 50% replacement ratio.

**Figure 16:**
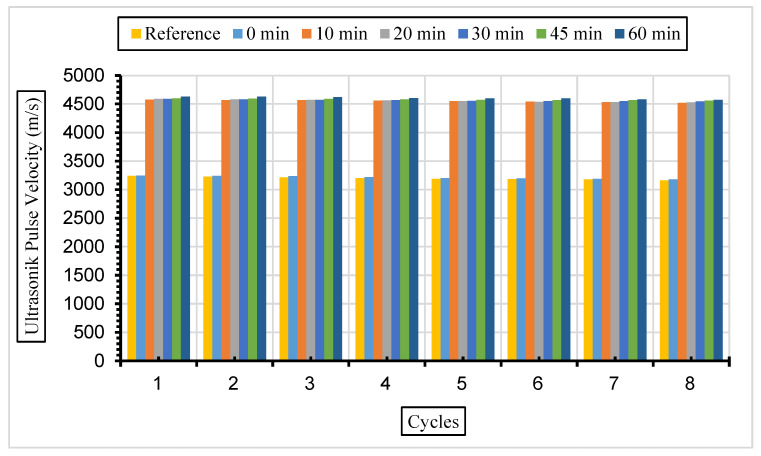
UPV values of concretes of different fineness values and 10% fly ash substituted in a 10% Na_2_SO_4_ solution.

**Figure 17:**
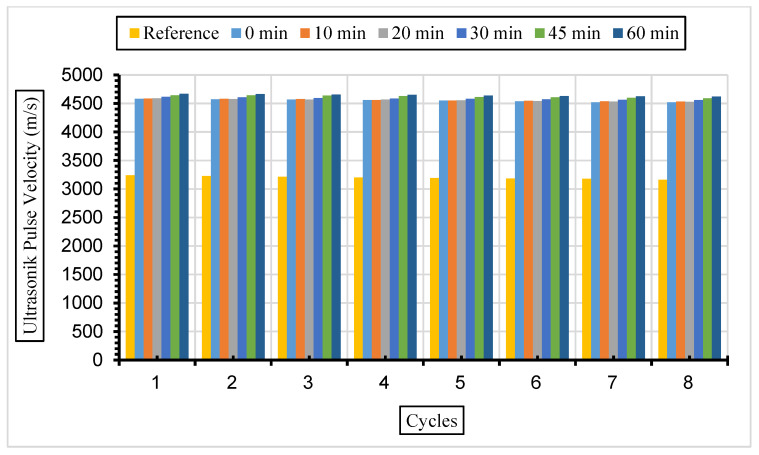
UPV values of concretes of different fineness values and 30% fly ash substituted in a 10% Na_2_SO_4_ solution.

**Figure 18:**
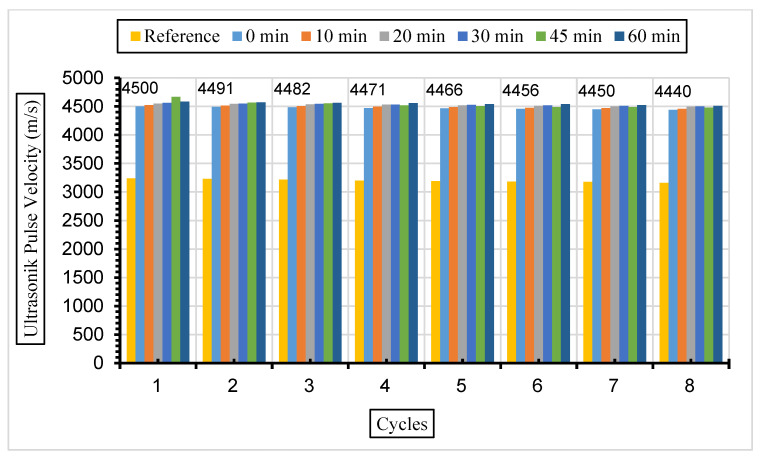
UPV values of concretes of different fineness values and 50% fly ash substituted in a 10% Na_2_SO_4_ solution.

After the same samples were exposed to a 10% Na_2_SO_4_ solution, ultrasonic velocity values were obtained for eight cycles, and the results are given in Figure 16, Figure 17 and Figure 18.

In this study, it was determined that the ultrasonic pulse velocity values increased as a result of the increase in fineness of the fly ash with grinding. Moreover, it was observed that there was a decrease in ultrasonic wavelength velocities by increasing the fly ash replacement ratio gradually (10%, 30%, and 50%). The ideal substitution rate was determined to be 30%, and the highest ultrasonic pulse velocity values were obtained at this substitution rate. It has been understood that the ideal ratio among the fly ash replacement rates used instead of cement in concrete is 30%. Ultrasonic velocity values were found to decrease with increasing sulphate amounts. It has been revealed that the increase in the fly ash substitution rate in the concrete fills the voids better and increases the strength and durability of the concrete. Liu et al. [80] exposed the concrete they produced using 0%, 20%, and 40% fly ash substitution to a 5% sodium sulphate solution in their study. According to their results, it was observed that the highest ultrasonic wavelength was in the concrete with 40% fly ash replacement. Aydın and Balkis [81] noted in their study that the use of fly ash in concrete contributed to the formation of the C-S-H structure, which contributes to the strong and durable structure of the concrete. The C-S-H structure formed by fly ash causes it to fill the gaps and form an impermeable material. An increase in fly ash causes a decrease in the pore structure and number of pores formed while also causing an increase in the ultrasonic impact velocity and compressive strength of the concrete. Additionally, high ultrasonic pulse velocity and low weight loss values were obtained in mixtures containing 40% fly ash.

Rao and Kumar [82] subjected several samples to several tests to determine the mechanical and durability properties of concrete prepared using fly ash instead of cement at 0, 5%, 20%, 40%, and 60% replacement rates. In these test results, they achieved the optimum replacement rate at 40% fly ash, and they found that the increase in fly ash substitution reduced the void volume and increased the ultrasonic pulse velocity values.

It is stated that when average fly ash is used in concrete, porosity decreases as it fills the spaces between aggregates [83]. The decrease in porosity value with the use of fly ash provides an increase in the ultrasonic wave speed. It has been observed that fly ash with a fine particle size prevents water passage by filling the spaces between cement particles. This makes concrete or mortar with fly ash additives more resistant to acid damage [84]. Moreover, it strengthens the weak points of old mortar or concrete by creating a micro-aggregate effect [85]. It was observed that the mixtures containing fly ash caused the ettringite formation to remain stable compared to the mixtures with ordinary Portland cement. In the presence of excessive aluminium in the environment, ettringite was converted to monosulphate, enabling the concrete structure to gain strength against many negative factors [86]. In many studies, using fly ash instead of cement in concrete, whether in different sizes or at different replacement rates, produces more durable and long-lasting concrete because of the physical, chemical, and mechanical effects of concrete. According to the results obtained in this study, it was seen that increasing both the thinness and the substitution rate caused an increase in the ultrasonic speed values.

## 4. Conclusions

After grinding the fly ash for 0, 10, 20, 30, 45, and 60 min, admixed concretes were produced by substituting 10%, 30%, and 50% of the cement with fly ash with different Blaine fineness values of 1555, 1632, 2347, 2589, 2766, and 3433 cm^2^/g, respectively. The compressive strength values determined after the 28-day curing period of the produced concrete were kept in 5% and 10% Na_2_SO_4_, the weight loss was determined after the application of eight cycles, and the ultrasonic pulse velocity values were determined. According to the results obtained,

1. It has been observed that the weight loss in fly ash-doped concrete has lower values than the reference concrete. The fineness of fly ash and the grinding effect reduced the weight loss values. In addition, the weight loss values increased due to the sulphate solution ratio to which the concrete was exposed and the increase in cycle time.
2. Grinding fly ash to a finer size better filled the voids and improved the concrete’s impermeability. Thus, the gaps in the concrete were filled, and higher results were obtained in compressive strength and ultrasonic pulse velocity values.
3. Compressive strength losses occurred as the admixture rate increased in fly ash-added concrete samples with different fineness values. The highest to lowest compressive strength values were observed in concretes with 30%, 10%, and 50% fly ash replacement, respectively.
4. According to the fly ash substitution rate, the highest weight loss in concrete samples exposed to a 5% Na_2_SO_4_ solution was observed in concrete samples with 50% substitution and the lowest in concrete samples with 30% substitution. The reason is that using waste material such as fly ash in concrete at a high value, such as 50%, has caused an increase in pressure loss and weight loss.
5. Compared to the samples kept in a 5% Na_2_SO_4_ solution, lower ultrasonic pulse velocity data were obtained. However, the decreases in the ultrasonic pulse velocity experiments obtained after the increase in the solution could be more apparent.
6. Blaine fineness values of 1555, 1632, 2347, 2589, 2766, and 3433 cm^2^/g were obtained after grinding fly ash for 0, 10, 20, 30, 45, and 60 minutes, respectively. The percent increase in Blaine fineness values after grinding fly ash at different times was calculated, and the effect of fineness was evaluated. The increase in grinding time on the fineness value was calculated as 4.95%, 50.93%, 66.49%, 77.88%, and 120.77%, respectively. This value positively affected the study’s weight loss, compressive strength, and ultrasonic pulse velocity values. However, in the experimental studies obtained before and after the maximum grinding of fly ash, the desired results were obtained in the values of the experimental results when the substitution rate was used in low and medium amounts. For example, a fly ash substitution of 50% in the mixture caused a decrease in the values of the experimental results. If the grinding time was kept longer and the fly ash was thinned, the weight losses could be lower, and the pressure and ultrasonic pulse velocity values could be higher in concrete with a 50% fly ash substitution. Thus, fly ash, a natural waste product, could be utilized at a higher rate in concrete.
7. It was observed that the increase in fly ash fineness increased the pressure and ultrasonic pulse velocity values and decreased the weight loss values in all data. These values decreased depending on the increase in the amount of Na_2_SO_4_ solution to which the concrete was exposed. Optimum test results were seen in concrete samples with 30% replacement fly ash. The ideal replacement rate, pressure, ultrasonic pulse velocity value, and minimum weight loss were found at 30%, 10%, and 50% replacement rates, respectively. It has been observed that very high and very low fly ash waste rates in concrete do not increase the properties of concrete.
8. This study analysed the Blaine values of fly ash as a substitute for cement and showed that it has a preventive effect, particularly on sulphate reactions that damage concrete. According to these results, an increase in concrete performance is achieved, allowing the construction of more economical and sustainable structures.

Further work could include the replacement of cement with ash, considering different densities and finenesses, and adjusting the aggregate amount accordingly to maintain a consistent mix volume. However, it would also be appropriate to conduct studies with different properties and types of fly ash (especially F-type fly ash).

## Figures and Tables

**Table 1:** Chemical and physical properties of the cement.

Chemical Analysis (%)		Physical Tests	
SiO_2_	16.76	Thinness (45 μm over the screen. %)	8.50
Al_2_O_3_	3.27	Specific Gravity (g/cm^3^)	3.01
Fe_2_O_3_	3.12	Blaine (cm^2^/g)	4099
CaO	60.25	Initial Setting (hours-minutes)	2 h 45 min
MgO	1.75	Final Setting (hours-minutes)	3 h 30 min
SO_3_	2.70	Volume Expansion (mm)	0.9
Loss of ignition	5.27	Water Requirement %	29.2
Na_2_O	0.12		
K_2_O	0.62		
CI	0.0171	**Compressive Strength** **(N/mm^2^)**	
Unmeasurable	5.773	2 day	24.0
Free CaO	0.35	28 day	51.86
Total	100		

**Table 2:** Chemical and physical properties of the fly ash.

Chemical Analysis		Physical Tests	
Component, %	Fly Ash	Physical Properties	Components
SiO_2_	29.20	Thinness (45 μm over the screen. %)	50.72
Al_2_O_3_	11.50	Specific Gravity (g/cm^3^)	2.63
Fe_2_O_3_	6.54	Specific Surface (cm^2^/g)	1867
CaO	37.44	Initial Setting (hours-minutes)	2 h 45 min
MgO	1.80	Final Setting (hours-minutes)	3 h 20 min
Na_2_O	0.42	Volume Expansion (mm)	0
K_2_O	0.65	Water Requirement (%)	27.7
SO_3_	4.55	Water Content (g)	138.5
Cr_2_O_3_	0.05	**Blaine Test** (cm^2^/g)	
Mn_2_O_3_	0.05		
P_2_O_5_	0.45	0 min (Uncrushed) 10 min	1555
TiO_2_	0.50		1632
ZnO	0.001	20 min 30 min	2347
Loss of ignition	2.24		2589
Free CaO	4.61	45 min	2766
Total	100.00	60 min	3433

**Table 3:** Different specific surface area values for the fly ash.

Grinding Time of Fly Ash, min	Blaine Fineness, cm^2^/g
0 (Original Fly Ash)	1555
10	1632
20	2347
30	2589
45	2766
60	3433

**Table 4:** Proportions of the concrete mixture.

Type of Materials	Fine Aggregate	Medium Aggregate	Coarse Aggregate	w/c	Water (kg/m^3^)	Cement (kg/m^3^)	Fly Ash (kg/m^3^)
	(0–4 mm)	(4–11.2 mm)	(11.2–22.4 mm)				
Reference	686 kg/m^3^	328 kg/m^3^	801 kg/m^3^	0.55	190	348	0
10% Fly ash substitution	686 kg/m^3^	328 kg/m^3^	801 kg/m^3^	0.55	190	313.2	34.8
30% Fly ash substitution	686 kg/m^3^	328 kg/m^3^	801 kg/m^3^	0.55	190	243.6	104.4
50% Fly ash substitution	686 kg/m^3^	328 kg/m^3^	801 kg/m^3^	0.55	190	174	174

## Data Availability

All data generated or analysed during this study are included in this published article.
